# Challenges and Triumphs: Unusual Presentation of Tuberculosis in the Cuboid Bone Successfully Managed Through Surgical and Medical Intervention

**DOI:** 10.7759/cureus.53796

**Published:** 2024-02-07

**Authors:** Hardik Patel, Sandeep Shrivastava, Aditya Pundkar, Ankit M Jaiswal, Saksham Goyal

**Affiliations:** 1 Department of Orthopaedics, Jawaharlal Nehru Medical College, Datta Meghe Institute of Higher Education and Research (Deemed to be University), Wardha, IND

**Keywords:** osteoarticular tuberculosis, extrapulmonary tuberculosis, tuberculosis osteomyelitis of foot, foot tuberculosis, cuboid tuberculosis

## Abstract

This case report describes the unusual presentation of tuberculosis (TB) affecting the cuboid bone in a 16-year-old male patient. The patient presented with a one-year history of progressive foot pain, a discharging sinus, evening rise of temperature, weight loss, and loss of appetite. Clinical examination revealed soft tissue swelling and the presence of caseous material oozing from the sinus. Emergency debridement and curettage were performed, and bone cementing was carried out. An intraoperative sample was sent for a culture sensitivity test, histological analysis, and cartridge-based nucleic acid amplification test (CBNAAT). Histopathological examination, CBNAAT, and culture and sensitivity tests confirmed the diagnosis of *Mycobacterium tuberculosis* infection. Post-operatively, anti-tuberculous treatment was started. The patient fully recovered from TB of the cuboid.

## Introduction

Among the serious health issues that developing nations have to deal with, tuberculosis (TB) stands out. Globally, around 27.1% of patients suffering from TB are from India. About 10% of instances of extrapulmonary TB are of the musculoskeletal variety. Foot TB appears to be a rare clinical condition, accounting for 0.1-0.3% of extrapulmonary TB and less than 10% of osteoarticular TB. Isolated TB of the cuboid is an extremely rare disease with very few case reports worldwide (with an incidence rate of less than 0.05%). In all, 1-3% of patients with TB have skeletal involvement [1]. It has been observed that the spine and extraspinal articulating joints are the site of genesis for 51% of skeletal TB [1]. Musculoskeletal cases of extrapulmonary TB account for about 10% of cases [2]. Foot TB appears to be a rare clinical condition that accounts for 0.1-0.3% of extrapulmonary TB and less than 10% of osteoarticular TB [3]. The most frequently affected areas in TB foot are the calcaneus and tarsal joints, then the talus, distal end of the first metatarsal, navicular, cuneiforms, and cuboid bones [3]. Isolated TB of the cuboid is a very rare presentation (<0.05%) for TB [3]. TB of joints has a prolonged onset, which is rarely diagnosed before it develops into the stage of severe arthritis [4].

Since non-destructive forms of joint inflammation are previously seen during the acute phase of TB, Poncet’s illness, also known as tuberculous rheumatism, is differentiated from TB arthritis. Poncet’s disease, also known as reactive arthritis, is a sterile arthritis that occurs as a reaction to an infection elsewhere in the body. It typically involves large joints, such as the knees, ankles, and wrists. Meanwhile, TB arthritis is caused by *Mycobacterium tuberculosis*, the same bacteria that causes TB in the lungs. It primarily affects weight-bearing joints, such as the hips and knees, and can also involve the spine (Pott’s disease). [5]. Synovitis-like symptoms are the first signs of TB arthritis, which progresses to periarticular demineralization, marginal erosions, and ultimately, joint destruction [4]. Patients who are overweight have shown a quicker progression from inflammation of the synovium to joint degeneration. A systemic inflammatory response is involved in the acceleration of joint degeneration in cases of superinfection (*Staphylococcus aureus*) [6].

The non-directional deceptive presentation of patients with malaise, anorexia, and other constitutional symptoms is a crucial factor in the delayed diagnosis of TB [7]. Delays in diagnosing the underlying cause of a mycobacterial infection and initiating therapy contribute to the loss of additional bone, adjacent bone, or joint tissue [8].

## Case presentation

A male patient, 16 years of age, presented to the AVBRH orthopedics outpatient department (OPD) with a one-year history of increasing foot discomfort and a discharging sinus over the dorsal aspect of his right foot. There was a history of decreased appetite, weight loss, and increased body temperature in the evenings for the last six months. The patient denied having ever experienced trauma. Moreover, there was no history of bronchial asthma, hypertension, diabetes mellitus, or TB. There is no history of substance abuse, ethnicity, or stays in refugee camps, prisons, or night shelters.

Examination of the right foot

Examining the right foot revealed a prominent soft tissue swelling across the dorsal side measuring 3 cm × 3.5 cm. The swelling was described as non-fluctuant, non-compressible, non-reducible, and non-pulsatile. There was a localized increase in temperature, no skeletal tenderness was felt, and the fluctuation test came out negative. There were no arterial bruits or venous hum on auscultation. A discharging sinus of 0.5 × 0.5 cm in size was noted, and clinical inspection revealed the presence of caseous material oozing from the sinus. No regional lymphadenopathy was observed, but muscle wasting was present. Active ankle and toe movements were intact, distal circulation was intact, and capillary refill was present. The results of hematological studies showed that the C-reactive protein (CRP) value was 565 mg/L, the erythrocyte sedimentation rate (ESR) was 45 mm/h, and the total white cell count (TWC) was 8000/μL. There was no growth in the blood culture or sensitivity test.

**Figure 1:**
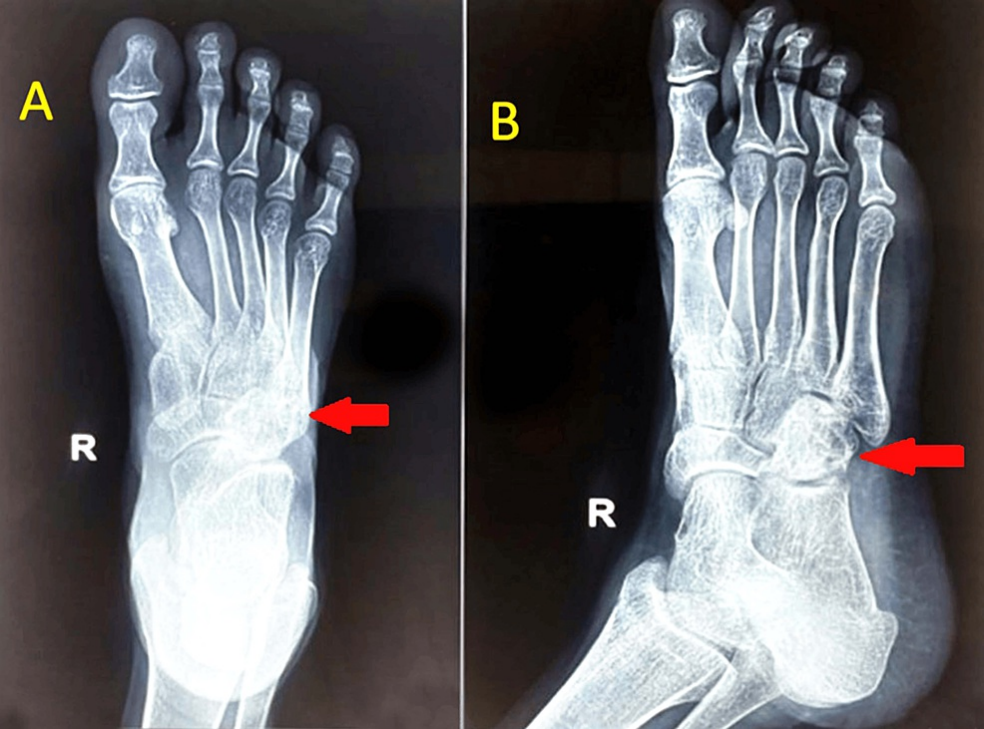
X-ray of the right foot in anteroposterior (A) and oblique (B) views showing an osteolytic lesion over cuboid bones along with soft tissue swelling and diffuse transient osteopenia

An X-ray of the right foot obtained during radiological studies showed diffuse osteopenia, soft tissue edema, and an osteolytic lesion over cuboid bones (Figure 1). The chest X-ray was normal.

**Figure 2:**
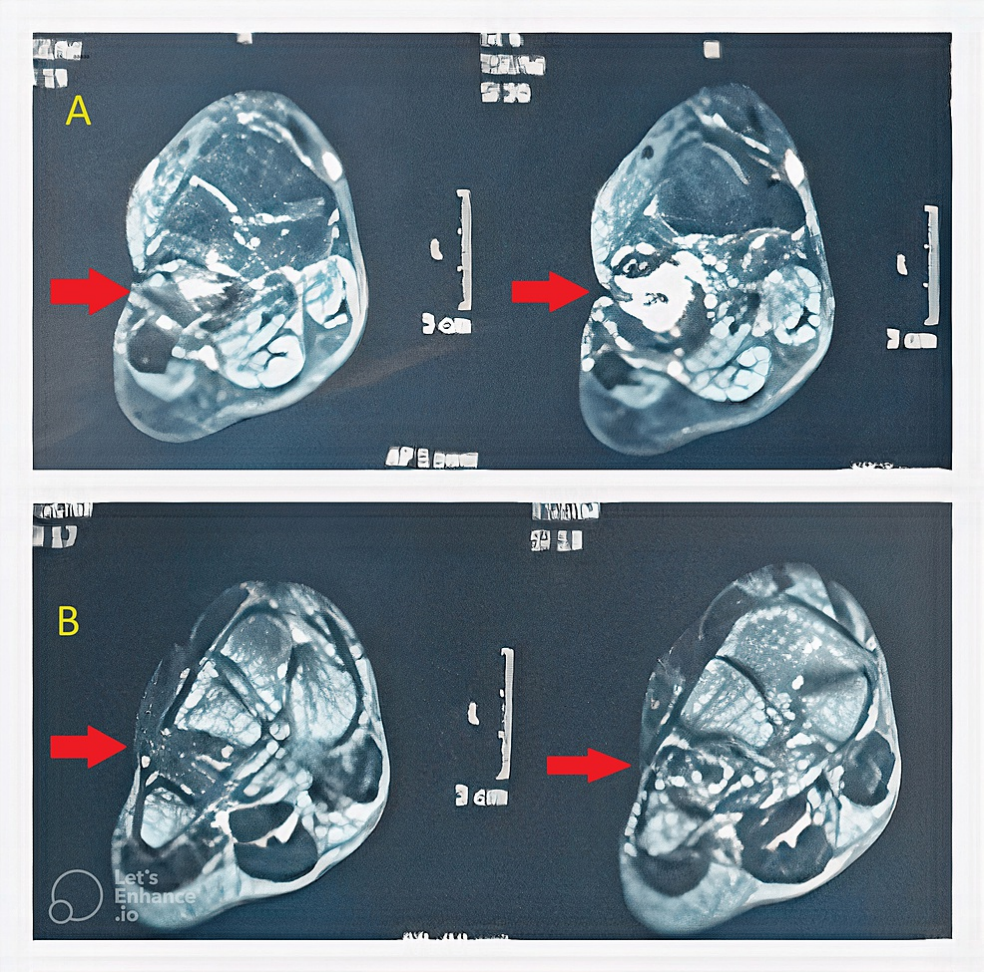
MRI of the right foot T2W (A) and T1W (B) images in the coronal view showing T1W hypointense signals and T2W hyperintense marrow signals involving the cuboid bone with an associated cortical break and sinus tract suggestive of osteomyelitis

On magnetic resonance imaging (MRI) of the right foot, T2W hyperintense marrow signals involving the cuboid bone with an associated cortical break and sinus tract were suggestive of osteomyelitis (Figure 2).

Surgical intervention

**Figure 3:**
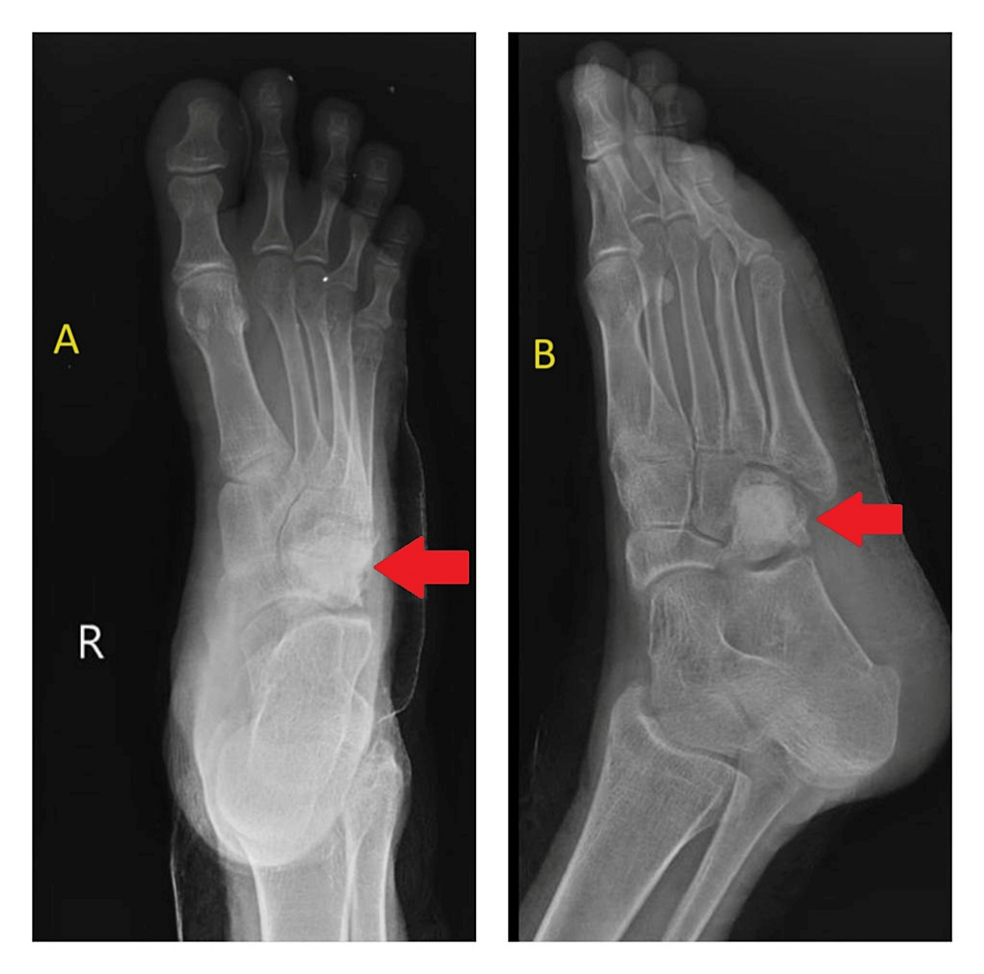
Post-operative X-ray of the right foot in anteroposterior (A) and oblique (B) views showing that the cuboid bone was filled with bone cement, the infection was resolved, and the cuboid lesion was consolidated

The patient underwent debridement and curettage to remove the infected tissue and caseous material, along with excision of the sinus tract. The adjacent intertarsal joints were affected. Antibiotic-impregnated bone cementing (4 g of vancomycin was used in 40 g of bone cement) was subsequently performed to stabilize the affected cuboid bone and promote healing (Figure 3).

Intra-operatively, a thick, solid, compressed caseous material was observed and noticed. Intra-operative specimen samples were sent for microbiological examination and cartridge-based nucleic acid amplification test (CBNAAT). The pus culture and sensitivity tests were positive for methicillin-sensitive *S. aureus* and *M. tuberculosis*. The patient was put on an injection of ceftriaxone + sulbactam 1.5 g intravenously every 12 hours based on the culture and sensitivity data. The CBNAAT report came back positive for rifampicin-sensitive *M. tuberculosis*. The patient was initiated on an anti-TB (AKT-4) regimen for 12 months and was subsequently discharged. The patient returned for a follow-up after three months of AKT. After the initiation of the AKT regimen, inflammation and pain reduced significantly, and the wounds dried with no discharge. Blood investigations were repeated every month for six months and once at the end of nine and 12 months. After completion of 12 months of AKT-4, the end of the results of hematological studies revealed that the CRP value was 260 mg/L, the ESR was 10 mm/h, and the total TWC was 5500/μL, all within normal limits.

## Discussion

The presented case of an unusual manifestation of TB in the cuboid bone underscores the challenges posed by atypical presentations of this infectious disease. The successful management, achieved through a combination of surgical and medical interventions, brings attention to the complexity of such cases and highlights the importance of a multidisciplinary approach [9]. The initial challenge lay in recognizing and diagnosing TB in an uncommon location, such as the cuboid bone. The patient’s symptoms, including progressive foot pain, a discharging sinus, and constitutional symptoms like weight loss and evening rise in temperature, were suggestive of an infectious etiology. However, no history of trauma demanded a full diagnostic workup [3]. The diagnostic journey involved a series of investigations, including histopathological examination, CBNAAT, culture sensitivity tests, and radiological studies. The confirmation of *M. tuberculosis *infection and the identification of co-infection with *S. aureus* added layers of complexity to the case. A superimposed staphylococcal infection on a clavicle infected with TB is a rare presentation, with only a few cases reported to date [10]. This highlights the importance of thorough microbiological examination to tailor the treatment plan effectively [11].

Surgical intervention, consisting of debridement, curettage, and bone cementing, was pivotal in removing the infected tissue, promoting bone healing, and preventing further spread of the infection. The intraoperative discovery of a thick, solid, compressed caseous material emphasized the severity of the infection and the need for prompt and thorough intervention [12]. The medical management, guided by the culture and sensitivity reports, involved a combination of intravenous antibiotics targeting *S. aureus* and the initiation of an anti-TB regimen. The successful outcome, as evidenced by the reduction in inflammation, pain, and complete healing of the wound, underscores the effectiveness of the chosen interventions [13]. This case prompts a reflection on the importance of considering TB in unusual anatomical locations, especially when faced with persistent symptoms and diagnostic uncertainty. The collaboration between orthopedic surgeons, infectious disease specialists, and microbiologists played a crucial role in achieving a positive outcome. Additionally, the successful management of co-infections emphasizes the need for a comprehensive approach to address multiple pathogens simultaneously [14].

In developing countries, TB has long been a serious infection. For the presentation of a wide range of pathologies of osseous TB, there are several clinical and radiographic features and findings. Therefore, it is difficult and challenging to diagnose the involvement of extrapulmonary features in TB [1]. Additionally, the lesions may be mistaken as chronic osteomyelitis, which similarly exhibits bone damage. When treating a case of bone destruction, debridement of the bone is used to assist in eradicating the infection, and if a defect is found, an antibiotic cement spacer is used [15]. The instance that was just mentioned is that of a foot abscess. If a concurrent infection is discovered prior to diagnosis, the antibiotic-impregnated medium proves to be successful in treating the local infection at the site by delivering a high concentration of antibiotics [16]. The presentation of TB in small bones is very rare, and because there is little reason to suspect the disease, the diagnosis is typically made later. Osteoarticular TB ranks fourth in extrapulmonary TB, behind urogenital tract, ganglionic localization, and vertebral TB. The vertebrae alone are the most common location [1]. The gateways for extrapulmonary TB are primary foci reactivation and secondary dispersion through the blood circulation [17].

As per the gold standard of investigation for the confirmation of TB, the histological pattern of any body fluid or tissue specimen is needed to find acid-fast bacillus (AFB) [18]. When bone scans are done, an enhanced uptake is seen, but this is not particular. MRI is an additional tool that can be used to demonstrate synovial inflammation, fluid accumulation, bony erosions, and bone lesions. Therefore, MRI also helps to reveal the severity of the disease, and MRI can be repeated for the follow-up of the progression of severity, though MRI is non-specific [19]. Tuberculin skin test (TST) or Mantoux TST is a supportive diagnostic test. Interferon-gamma release assays, such as QuantiFERON-TB Gold, are specific for identifying tuberculin infection but not for differentiating between latent TB infection and active disease [20]. For the detection of amplified TB DNA, tests using polymerase chain reaction are extremely sensitive, but this test is unable to differentiate between live and dead bacilli. The polymerase chain reaction test is a more precise and quick diagnostic method for the examination of synovial fluid, bone, and soft tissue in joints [21]. The treatment regimen for tubercular arthritis is easy from the classic covers of the care of osteoarticular TB, in which only chemotherapy is required. Nowadays, chemotherapy has been exchanged over an increasingly shorter treatment program of six months by using far more efficient drugs for treatment [22]. Following the application of an orthopedic foot splint, therapy was carried out until the clinical signs subsided, which could take up to four weeks [23]. The patient was initiated on anti-Koch’s treatment (AKT) for 12-18 months, a standard extrapulmonary TB treatment regimen comprising isoniazid, rifampicin, ethambutol, and pyrazinamide for two months followed by isoniazid, rifampicin, and ethambutol for 10-16 months [24]. To date, treatment options for this condition are still controversial. Surgical debridement is the debatable option of treatment for small bone TB, and the period of antitubercular chemotherapy is an unnoticeable issue of discussion. Chemotherapy must be administered prior to any kind of surgical debridement, even if antitubercular chemotherapy and debridement are chosen as the course of treatment, in order to minimize bone loss and disease propagation. Antitubercular chemotherapy should be administered for 12-18 months [25].

In our case report, atypical site of occurrence, clinical manifestation, and delay in biopsy lead to delayed diagnosis of cuboid TB. Early diagnosis would have allowed anti-tubercular chemotherapy to be started as soon as symptoms appeared, reducing the need for surgical debridement and improving patient outcomes earlier.

## Conclusions

Overcoming the challenges in diagnosing and treating TB in the cuboid bone showcased resilience and expertise, resulting in a successful outcome. This case contributes valuable insights to medical literature, highlighting the complexities of managing atypical TB presentations through a multidisciplinary approach. The successful management involved a comprehensive strategy of surgical debridement, bone cementing, and medical intervention. Meticulous investigations and multidisciplinary collaboration addressed the complexities of *M. tuberculosis* and *S. aureus* co-infection. Surgical intervention eliminated the infected tissue and stabilized the bone, complemented by tailored medical management that led to a positive outcome with reduced inflammation and complete wound healing. This case underscores the significance of adaptability and collaboration in effectively addressing atypical infectious disease presentations.
